# Questionnaire survey for the International Journal of Legal Medicine on the occassion of the 26th triennal meeting of the International Academy of Legal Medicine

**DOI:** 10.1007/s00414-024-03310-3

**Published:** 2024-08-28

**Authors:** Andreas Schmeling, Tony Fracasso

**Affiliations:** 1 Institute of Legal Medicine, Münster, Germany; 2 Centre Universitaire Romand de Médicine Légale (CURML), Geneva, Switzerland

**Keywords:** Questionnaire, Guidelines, Publication ethics, Reviewer recruitment

## Abstract

A questionnaire was prepared in advance of the 26th triennial conference of the International Academy of Legal Medicine (IALM) and sent to 474 email addresses included in the IALM mailing list. The questionnaire addressed three current challenges faced by the International Journal of Legal Medicine (IJLM): the publication of guidelines and validation studies in the field of legal medicine, the publication ethics of case reports, and the recruitment of new reviewers for the IJLM. The response rate was 20%. The survey results highlight the need for international guidelines in various areas of legal medicine. Some desired guidelines already exist. To provide visibility and knowledge of the existing national guidelines, the IJLM has launched a Topical Collection on Quality Assurance in Legal Medicine. This collection aims to inform readers about country-specific characteristics of legal medicine structures and the existing national guidelines.Around 80% of the participants stated that there are legal or ethical requirements for the publication of forensic case reports or case series. Various options for obtaining consent for publication are discussed. Eighty-six of the 97 participants indicated their willingness to review manuscripts for the IJLM. It is emphasized that the contributions of reviewers should be duly recognized and valued.

## Introduction

On the occasion of the General Assembly of the International Academy of Legal Medicine (IALM) held in Strasbourg on June 1, 1994, the International Journal of Legal Medicine (IJLM) was designated as the official journal of the IALM [20]. In December 2022, IJLM celebrated its centenary. To mark this milestone, the Editors of the Journal decided to publish a series of four articles analyzing the evolution of legal medicine through more than 8,000 articles published over a century [14, 21, 24, 25].

One of the most notable developments in recent decades is the increasing importance of disciplines previously considered ancillary, such as forensic toxicology and genetics. Additionally, new significant topics have emerged, including age estimation, forensic anthropology, forensic imaging, and forensic entomology [14]. The analysis of the published articles revealed that forensic toxicologists, geneticists, anthropologists, entomologists, and age estimation experts have worked intensively within their specialist communities to develop a common knowledge base and recommend best practices in their sub-disciplines. In contrast, forensic pathologists have often taken an individual, inconsistent approach, influenced by specific local opportunities or personal interests. As a result, internationally validated and recognized methods for estimating time since death, determining the age of a skin wound, and other tasks of forensic pathology do not exist.

Therefore, as suggested by Ferrara et al. [12], multicenter research groups should be established to conduct robust validation studies with a sufficient number of cases. Furthermore, national and international medicolegal societies should pool their expertise and resources to produce recommendations in key areas of interest [14].

In this context, a questionnaire was developed in preparation for the 26th IALM Conference held from May 21–23, 2024, in Athens. As editors-in-chief of the IJLM, we aimed to address three current challenges of the journal through this questionnaire:

- The publication of guidelines and validation studies in the field of legal medicine.
- Publication ethics of case reports and case series.
- The recruitment of new reviewers for IJLM.

The questionnaire sought to gather the experiences and opinions of IALM members on these challenges.

## Materials and methods

The questionnaire consisted of four parts with a total of 17 questions. In the first part (questions 1–3), participants were asked about the countries in which they work, the duration of their professional activity, and their specific field within legal medicine. Part 2 (questions 4–12) focused on national societies for forensic sciences and their specialized working groups, national guidelines, recommendations, or protocols, as well as the need for international guidelines, protocols, or validation studies. In the third part (questions 13–14), the national legal and ethical requirements for publishing case reports and case series regarding living and deceased individuals were explored. The fourth part (questions 15–17) inquired about the participants’ willingness to review manuscripts for the IJLM. Table 1 shows the survey questions.

**Table 1:** Questions of the survey

In which country are you active?
Which are the areas of legal medicine in which you are active? (More than one answer possible.)
How long have you been working in legal medicine?
Is there a society for forensic sciences in your country?
If so, which one(s)?
Are there specialized working groups in your country focusing on specific areas of legal medicine?
If so, which one(s)?
Are there any existing guidelines, recommendations, or protocols related to medicolegal topics in your country?
If so, which one(s)?
While clinical disciplines have numerous guidelines and recommendations, legal medicine lacks internationally validated guidelines and protocols. Do you believe there is a need for international guidelines and protocols in this field?
Which areas do you think are currently lacking guidelines, recommendations, or protocols?
Which topics do you believe lack validation studies?
Are there any legal or ethical requirements in your country for the publication of forensic case reports or case series regarding living individuals (e.g. victims of violence)? If so, what are the specific requirements (e.g. consent to participate, consent to publish, ethical approval)? Are there specific laws regarding this matter?
Are there any legal or ethical requirements in your country for the publication of forensic case reports or case series regarding deceased individuals or material/data collected during postmortem investigations? If so, what are the specific requirements (e.g. consent to participate of the relatives or clients, consent to publish of the relatives or clients, ethical approval)? Are there specific laws regarding this matter?
Are you available to serve as a reviewer for the IJLM?
If so, could you specify the topics you feel most competent in reviewing.
If you are available to serve as a reviewer, please provide your academic title, first name, surname, and email address for contact purposes.

An online version of the questionnaire was created using Google Forms. On November 27, 2023, an email was sent to 474 individuals on the IALM mailing list, inviting them to participate in the survey. The deadline for participation was set for December 31, 2023. On December 21, 2023, a reminder email was sent, extending the participation deadline to January 15, 2024.

**Table 2:** Countries in which the participants work

Country	Number of particpants
Italy	11
Germany	7
Spain	7
India	6
Switzerland	6
France	5
Romania	5
Belgium	4
Japan	4
Turkey	3
United Kingdom	3
Greece	2
Hungary	2
Portugal	2
South Africa	2
Taiwan	2
United Arab Emirates	2
USA	2
Argentina	1
Australia	1
Brazil	1
China	1
Croatia	1
Czech Republic	1
Denmark	1
Georgia	1
Indonesia	1
Iraq	1
Ireland	1
Kingdom of Saudi Arabia	1
Mexico	1
Morocco	1
Netherlands	1
Panama	1
Poland	1
Senegal	1

**Table 3:** Areas of legal medicine in which the participants are active

Area of legal medicine	Number of particpants
Forensic pathology	66
Clinical legal medicine	59
Medical malpractice	37
Forensic anthropology	36
Medical law and ethics	27
Traffic medicine	26
Forensic toxicology	21
Forensic imaging	17
Insurance medicine	9
Forensic genetics	6
Forensic entomology	2
Others	15

**Table 4:** Duration of professional activity of the participants

Duration of activity (in years)	Number of particpants
1–5	6
6–10	14
11–15	13
16–20	20
21–25	7
26–30	14
31–35	11
36–40	6
41–45	3
46–50	2

## Results

Ninety-seven participants completed the questionnaire, resulting in a response rate of 20%. Table 2 lists the countries in which the participants are active. Countries with more than five participants include Italy, Germany, Spain, India, and Switzerland. Table 3 outlines the main areas of activity of the participants, with most being active in forensic pathology, clinical legal medicine, forensic anthropology, medical malpractice, medical law and ethics, traffic medicine, and forensic toxicology. Table 4 details the duration of the participants’ professional activities, showing that most have been working in legal medicine for between 6 and 35 years.

Eighty-eight out of 97 participants reported the presence of a society for forensic sciences in their country. Sixty-eight indicated the existence of specialized working groups focusing on specific areas of legal medicine in their country. Seventy-seven participants stated that their country has guidelines, recommendations, or protocols related to medicolegal topics, with selected guidelines listed in Table 5. Ninety-one participants saw a need for international guidelines and protocols in legal medicine, with selected missing guidelines shown in Table 6 and selected missing validation studies in Table 7.

**Table 5:** Selected answers concerning exisiting national guidelines

External examination of bodies
Estimation of PMI
Forensic autopsy
Exhumation
DVI
Clinical forensic medicine
Forensic age estimation
Toxicological analyses
DNA analyses
Forensic Anthropology
Forensic Entomology

**Table 6:** Selected answers concerning currently lacking guidelines

Forensic pathology and histopathology
Forensic imaging
Forensic photography
Forensic toxicology
Forensic genetics
Forensic anthropology
Clinical forensic medicine
Forensic nursing
Forensic anthropology
Personal injury assessment
Forensic odontology
Mass disaster guidelines
Competency and proficiency testing

**Table 7:** Selected answers concerning currently lacking validation studies

Drowning
Hypothermia
Head injury
Asphyxia deaths
Wound vitality and wound age
Postmortem biomarkers
Postmortem interval estimation
Malpractice interpretation
Personal damage quantification
Correlations between blood drugs’ doses and effects
Evaluation of drug related death, specially in synthetic drugs
Age estimation
Ancestry estimation

Regarding the publication of forensic case reports or case series involving *living individuals*, 75 participants stated there are legal or ethical requirements in their country. Of these, 56 indicated that ethical approval, informed consent, or anonymization is required. Five participants mentioned the existence of a specific law, and one participant noted the use of informed consent forms when examining living individuals. Fourteen participants provided no further explanation, seven reported no such requirements in their country, and fifteen did not know or did not answer.

For the publication of forensic case reports or case series on *deceased persons*, 78 participants indicated legal or ethical requirements in their country. Of these, 57 cited the necessity of ethical approval, informed consent, or anonymization. Seven participants mentioned a specific law, and fourteen provided no further explanation. Eight participants reported no such requirements in their country, and eleven did not know or did not answer.

Eighty-six out of 94 responding participants expressed willingness to review manuscripts for the IJLM. For privacy reasons, the names and contact details of participants willing to act as reviewers for IJLM cannot be published.

## Discussion

### Participants of the survey

Surveys submitted to specific groups of professionals in order to get information on specific aspects of their activity have been repeatedly proposed in the forensic context in recent times [7, 9, 11, 19, 22]. We sent a questionnaire to 474 individuals on the IALM mailing list in order to gather the experiences and opinions of IALM members on current challenges of the IJLM. The response rate of our questionnaire survey was 20%, significantly below the average response rate for online surveys, which is 44% according to a meta-analysis by Wu et al. [26]. The primary reason for the comparatively low response rate is that the IALM mailing list, to which the survey invitation was sent, includes not only active IALM members but also resigned members, from whom responses were not necessarily expected. If only the number of active IALM members (*n* = 299) is taken into account, the response rate increases to 32%. However, this is still below the average response rate for online surveys.

There were more than five participants from Italy, Germany, Spain, India, and Switzerland. However, there were significant “blind spots” in Eastern Europe, Western and Northern Asia. Therefore, the results of the questionnaire survey are primarily valid for Western Europe, North and South America, and East Asia.

Most participants work in the fields of forensic pathology, clinical legal medicine, forensic anthropology, medical malpractice, medical law and ethics, traffic medicine, and forensic toxicology. Additionally, most participants have been active in legal medicine for between 6 and 35 years. Consequently, the results of the questionnaire survey are highly representative of this group of professionals.

### Guidelines and validation studies

The starting point for the questions on guidelines and validation studies was the finding from the analysis of articles published in the IJLM between 1990 and 2022 that there is a significant lack of guidelines and validation studies, particularly in the field of forensic pathology [14]. Ninety-one participants in our survey concurred that there is a need for international guidelines.

The participants listed numerous areas of legal medicine where they saw a need for guidelines. A comparison with existing national guidelines shows that such guidelines already exist for some of these areas in certain countries. However, the development of international guidelines in some areas is complicated by differences in legal systems and the organization of legal medicine between countries. To better understand these differences and to identify existing national guidelines, the IJLM has launched a Topical Collection on Quality Assurance in Legal Medicine. This collection aims to highlight country-specific characteristics of legal medicine structures and the existing national guidelines.

International guidelines are already available for some topics. The European Council of Legal Medicine (ECLM) has published collaborative papers on international standards and guidelines for forensic examinations by forensic physicians and pathologists. These guidelines cover areas such as the accreditation of pathology services, on-site scene and corpse investigation, examination of victims of sexual assault and elder abuse, and forensic teaching and training at the undergraduate and postgraduate levels [5, 8, 15, 17, 18]. We would strongly welcome further international guidelines published by the ECLM.

### Publication ethics of case reports

Many clinical journals require consent from patients, their guardians (in the case of minors), or next of kin (in the case of deceased individuals) for the publication of case reports or case series. Bonsu et al. [3] found a low level of declaration of informed consent in forensic science research publications. They examined the reporting of ethical approval and informed consent in original studies published in six forensic science journals from 2010 to 2019. In only 527 out of 3010 studies was informed consent said to have been sought. Of these, 155 reported that written informed consent was obtained, eleven stated oral consent, while the remaining 357 studies (67.7%) did not report the process used to gain consent.

It is important to note that the opportunities for medicolegal experts to obtain consent for publication differ significantly from those available to clinicians. Medicolegal experts operate outside the framework of a doctor-patient relationship; they function as neutral experts. Typically, the role of an expert witness does not allow for contacting the relatives of a living or deceased victim of violence to obtain consent for publication.

Given that ethical and legal perspectives can differ between countries, we included questions in our questionnaire about the national legal or ethical requirements for the publication of forensic case reports or case series regarding living and deceased individuals. Seventy-eight participants stated that there are legal or ethical requirements for the publication of forensic case reports or case series regarding deceased individuals in their country. Seventy-five participants answered affirmatively regarding the publication of case reports or case series about living individuals. One participant reported using forms to obtain consent to publish when examining living individuals.

In our view, the use of such forms, which can also include other relevant information for the clinical forensic examination and can be signed prior to the actual examination, is a good way to obtain informed consent. Appendix 1 shows an exemplary form that, in addition to consent to publication, also includes the use of biomaterials for research purposes.

In cases involving adults lacking the capacity to provide consent, minors, or deceased individuals, written consent to publish should be sought from the responsible party. This may include the next of kin, legal guardian, public prosecutor, or any other relevant authority involved in the medicolegal investigation. In instances where obtaining consent is not feasible, the case report should be anonymized to the greatest extent possible by excluding all information that could potentially reveal the identity of the individual described.

### Reviewer recruitment

Peer review is seen as the foundation of nearly all scientific publishing [10]. However, in recent years, journal editors have faced difficulties finding expert reviewers for the increasing number of manuscript submissions. This phenomenon has been termed the “reviewer crisis” [10, 23].

Ellwanger and Chies [10] analyzed the possible causes for reviewer refusal, identifying the proliferation of articles as the main reason. Landhuis [16] indicated that the number of published scientific papers had climbed by 8–9% per year over the past decades, with approximately two papers per minute being published in PubMed in the biomedical field alone. This trend of article proliferation is also evident in the IJLM. While an average of 39 articles were published per issue between 1970 and 1990, this figure rose to 121 articles per issue between 1990 and 2022 [14].

According to Baveye and Trevors [1], the article proliferation might be driven by the evaluation systems of universities and research centers that rely on scientometric indices, like the h-index, to evaluate the “impact” of their researchers, implicitly encouraging them to publish more articles. The globalization of research and the emergence of new research powerhouses, such as the People’s Republic of China and India, are also contributing to the increase in article numbers [2].

Fox et al. [13] noted that the rate of agreement to review declines as the number of review invitations received by an individual reviewer increases. Another factor associated with the refusal to review is the increasing amount of time researchers must spend on administrative tasks and fundraising. Additionally, dissatisfaction with scientific publishers is seen as a reason for reviewer refusal [10, 13].

Ellwanger and Chies [10] discussed potential solutions to the “reviewer crisis,” including the implementation of online platforms to register reviewer performance, careful selection of reviewers, and the provision of monetary and non-monetary rewards for reviewing. They also suggested recognizing top reviewers, formally acknowledging their contributions, linking reviewers’ names to published articles, and including young reviewers. Increasing diversity in the peer-review process in terms of gender, age, and geographic location of reviewers was also mentioned as a beneficial approach.

Tropini et al. [23] proposed increasing the use of artificial intelligence (AI) for tasks such as identifying plagiarism, image falsification, verifying correct statistical tests, ensuring proper language and grammar use, checking appropriate references, and confirming correct citation of figures. However, the most promising solution might be for universities and research institutions to include reviewer activities in the performance evaluation of scientists, similar to publications and third-party funding [1, 2]. Various reviewer indices have been proposed for this purpose [4, 6].

Of the 97 participants in our questionnaire survey, 86 agreed to review manuscripts for the IJLM. This result indicates a general willingness to serve as reviewers, a commitment that should be duly appreciated by the publisher. Currently, the names of reviewers are published once a year as an acknowledgment. Reviewer activities for the IJLM can be registered on the ORCID platform. Additionally, we will suggest that particularly active reviewers receive a certificate as a token of appreciation.

## Data Availability

Not applicable.

## Appendix 1: example of a consent form for the use of health data and biomaterials for research and publication

**Consent to the use of health data and biomaterials (tissue and body fluids) for medical research purposes and publications**.

1. I agree to the collection, processing, storage and scientific use as well as publication of my own health data / the health data of my child, if these data are inserted anonymously in publications and a traceability to my person / my child is not possible through the publication.

- Yes.
- No.

2. I consent to the collection, storage and analysis of the biomaterials from my body / the biomaterials from my child’s body (tissue, body fluids). I consent to the publication of the findings obtained from the analyses if the published data has been anonymized and it is not possible to trace the data back to me / my child.

- Yes.
- No.

The use of my anonymized health data and biomaterials / the anonymized health data and biomaterials of my child may take place within the framework of national and international projects. It may also include genetic testing of the samples.

My consent is voluntary and valid indefinitely. It can be revoked by me in full or in part at any time without giving reasons and without any disadvantages for me / my child. In the event of revocation, the biomaterials remaining for research and the data stored on the basis of this consent will be destroyed, insofar as this is legally permissible. Data from analyses already carried out can no longer be removed.

I have been informed about the use of my health data and biomaterials / my child’s health data and biomaterials and give my consent. I have had sufficient time to think about this and all my questions have been answered satisfactorily.

Place, date Signatures.
